# Application of integrated CRITIC and GRA-based Taguchi method for multiple quality characteristics optimization in laser-welded blanks

**DOI:** 10.1016/j.heliyon.2022.e11349

**Published:** 2022-11-01

**Authors:** Teerapun Saeheaw

**Affiliations:** Department of Teacher Training in Mechanical Engineering, King Mongkut's University of Technology North Bangkok, Bangkok, Thailand

**Keywords:** CRITIC, Grey relational analysis, Taguchi method, Weld strength, Weld width

## Abstract

There is a need to study the multi-objective optimization technique as the appropriate method to enhance welding qualities under optimal process conditions. Therefore, this study investigated the application of the integrated Criteria Importance Through Intercriteria Correlation (CRITIC), which is an objective weighting technique, and the Grey Relational Analysis (GRA)-based Taguchi method to solve multiple criteria optimization problem in the Nd:YAG laser welding process. The Taguchi-based L36 orthogonal array table was employed in this study to optimize six process parameters, including the beam diameter, laser power, flow rate, welding speed, laser offset, and pulse shape, with the aim to simultaneously achieve maximum weld strength and minimum weld width. The base metal JIS G3141 SPCC steel with 0.5 and 1.0 mm thicknesses was used in the present experiment. Following the welding process and optimization, the weld strength was measured using a Cometech QC-506M1 universal testing machine, while the weld width was determined under a Nikon SMZ25 stereomicroscope. Based on the results, the weight fractions of the weld strength and weld width from the applied CRITIC method were equal to 0.4157 and 0.5843, respectively. Meanwhile, the GRA revealed that the process parameters recorded an optimal setting for beam diameter of 0.8 mm, flow rate of 8 L/min, laser power of 0.6 kW, welding speed of 2.5 mm/s, laser offset of 0.2 mm, and pulse shape I. Furthermore, the weld strength and the weld width were enhanced from 236 to 328 MPa and from 1.13 to 1.04 mm, respectively. Additionally, the Analysis of Variance (ANOVA) indicated that the laser power and welding speed were the most influential parameters on the welding qualities. Most importantly, the findings of the confirmation experiment showed that the proposed approach was able to effectively identify the optimal laser welding parameters, which ultimately improved the multiple quality characteristics.

## 1 Introduction

Laser Welded Blanks (LWBs) or tailored blanks are semi-finished parts, which are produced by laser welding two sheets of identical or dissimilar material (depending on the application) typically prior to a forming process. LWBs in the body-in-white structure offers several significant advantages, including weight loss, low energy consumption, more environmentally friendly, and enhanced dimensional consistency.

Laser beam welding is a fusion joining process that involves the laser application to join two metal pieces together. The highly precise, reproducible, and minimum controlled heat input of the Nd:YAG pulsed laser beam has been successfully implemented in various applications that require reliable and outstanding performance in the field of electrical and electronics, medical, nuclear, aerospace, and petrochemical industries. Furthermore, pulsed laser welding is able to weld heat-sensitive materials and produces very little heat input to the workpiece, subsequently resulting in low distortion [1]. The weld quality, particularly the weld bead geometry and mechanical stability, is highly dependent on the material properties and the process parameters. Therefore, it is essential to identify the optimal process condition to improve the weld quality. The multi-objective optimization technique should be considered as the appropriate method to simultaneously evaluate the objective and process parameters.

The application of the Taguchi method involves a comprehensive experimental design and analysis of a product to formulate and improve its quality [2]. Over the years, the method has become a compelling instrument in the research and development sector to achieve high-quality products in a cost-effective and time-efficient manner. Previous studies have shown that the Taguchi method provides highly effective approaches for optimizing the processing conditions in many manufacturing processes [3, 4, 5, 6, 7]. Despite its impressive performance, the design of the traditional Taguchi method is limited to optimizing a single quality feature at a time. In contrast, the optimization of multiple quality characteristics is far more complex compared to that of a single quality characteristic [8, 9, 10]. On one hand, improving a specific quality characteristic could potentially cause a major degradation of other vital quality characteristics. On the other hand, applying all quality characteristics with the same weight would cause substantial yield loss since each quality characteristic may be essentially different.

Previously, Diakoulaki *et al.* [11] introduced the Criteria Importance through Intercriteria Correlation (CRITIC) method, which permits greater flexibility in terms of the scientific weight assignment based on the variation in the parametric values. The evaluation process, which eliminates human intervention, defines the weights according to the contrast intensity and conflict evaluation of the decision problem. Given the incapability of the conventional Taguchi method to solve multi-objective optimization problems, the grey system theory was adopted by Ju-Long [12] to develop the Grey Relational Analysis (GRA), which can be used to determine the optimal parameters and effectively solve complex interrelationships between multiple quality characteristics in various manufacturing processes [13, 14].

Although many researchers have performed single quality characteristic analyses, the single-objective approach is composed solely of sufficient simplifications of the real problem. Naturally, welding processes are complicated and often involve the optimization of numerous contradicting objectives. Conventionally, skilled operators choose parameters based on trial-and-error method which was time consuming for every new welded product to meet the required specification to the welded joint. In order to produce the desired quality weldments accurately without consuming time, materials and labor effort, the concept of Taguchi's orthogonal array is used to construct a limited number of experiments through a well-balanced design, and Taguchi's quality loss function is utilized to optimize the output responses carried out from the experiments. However, the optimal design of Nd:YAG laser welding process parameters could be difficult as more than one quality characteristic are used to represent the overall quality. Under these circumstances, GRA is introduced and conducted to develop a correlation between the process quality characteristics. Hence, in this study, the GRA-based Taguchi method is the preferred solution to determine the combined response parameters [13, 15, 16]. Nevertheless, the prospect of integrating the CRITIC and GRA-based Taguchi method for optimal Nd:YAG laser welding process with multiple quality characteristics has not been published.

**Figure 1:**
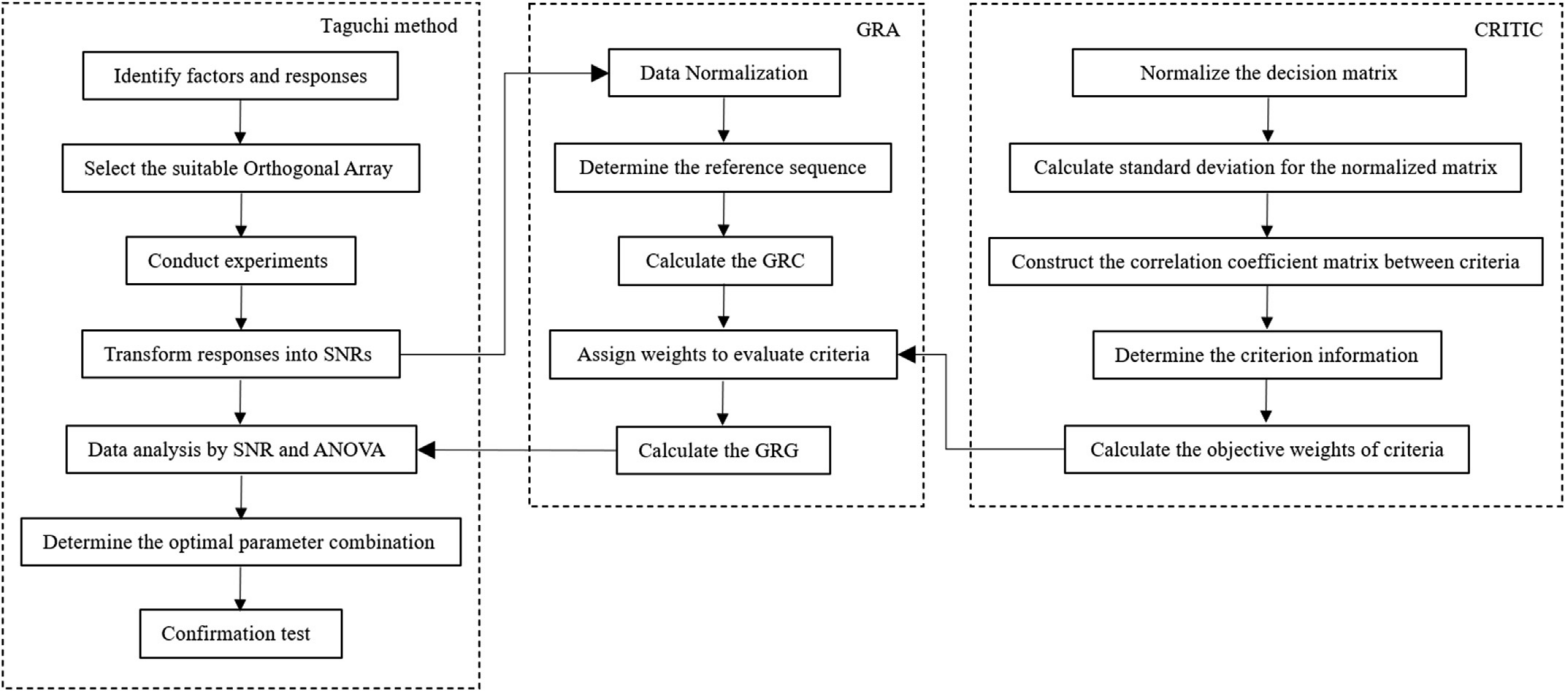
Flowchart of the integrated CRITIC and GRA-based Taguchi method.

Realizing this research gap, this study investigated the integrated CRITIC and GRA-based Taguchi method to optimize the Nd:YAG laser welding process under multiple weld qualities, including the weld strength and weld width. The flow diagram of the proposed optimization approach is illustrated in Figure 1. The welding trials were carried out using a Taguchi-based L36 orthogonal array with varying parameters comprising beam diameter, laser power, flow rate, welding speed, laser offset, and pulse shape. The single-objective optimization was carried out via Taguchi's Signal-to-Noise Ratio (SNR) analysis, followed by the GRA-based Taguchi method to evaluate the response of the multi-objective optimization. The weight fraction for the individual objective function was then determined by employing the CRITIC method. Furthermore, the GRA with CRITIC weighting was employed to convert the multiple quality characteristics into a single quality characteristic known as the Grey Relational Grade (GRG). Subsequently, the Analysis of Variance (ANOVA) was conducted to determine the impact of the laser beam welding processing parameters, followed by the verification of the optimum parameters with respect to the obtained multiple quality characteristics. Finally, a concise summary concludes the findings of this study.

## 2 Experiment

A low carbon steel sheet grade classified under the SPCC-type Japanese Industrial Standard (JIS) G3141, designated as the base metal JIS G3141 SPCC steel, was used in the present experiment and defined as commercial-grade cold-rolled steel. This steel is an ideal material for automobiles, electrical appliances, and other products due to its wider workable range. The chemical content (wt%) and mechanical properties of the work material are provided in Tables 1 and 2, respectively.

**Table 1:** Chemical content (wt%) of the base metal JIS G3141 SPCC steel.

Thickness (mm)	C	Si	Mn	P	S
0.50	0.0020	0.002	0.107	0.0101	0.0034
1.00	0.0028	0.002	0.116	0.0112	0.0034

**Table 2:** Mechanical properties of the base metal JIS G3141 SPCC steel.

Thickness (mm)	Yield Strength (MPa)	Tensile Strength (MPa)	Elongation (%)
0.50	169	258	38
1.00	195	286	37

**Figure 2:**
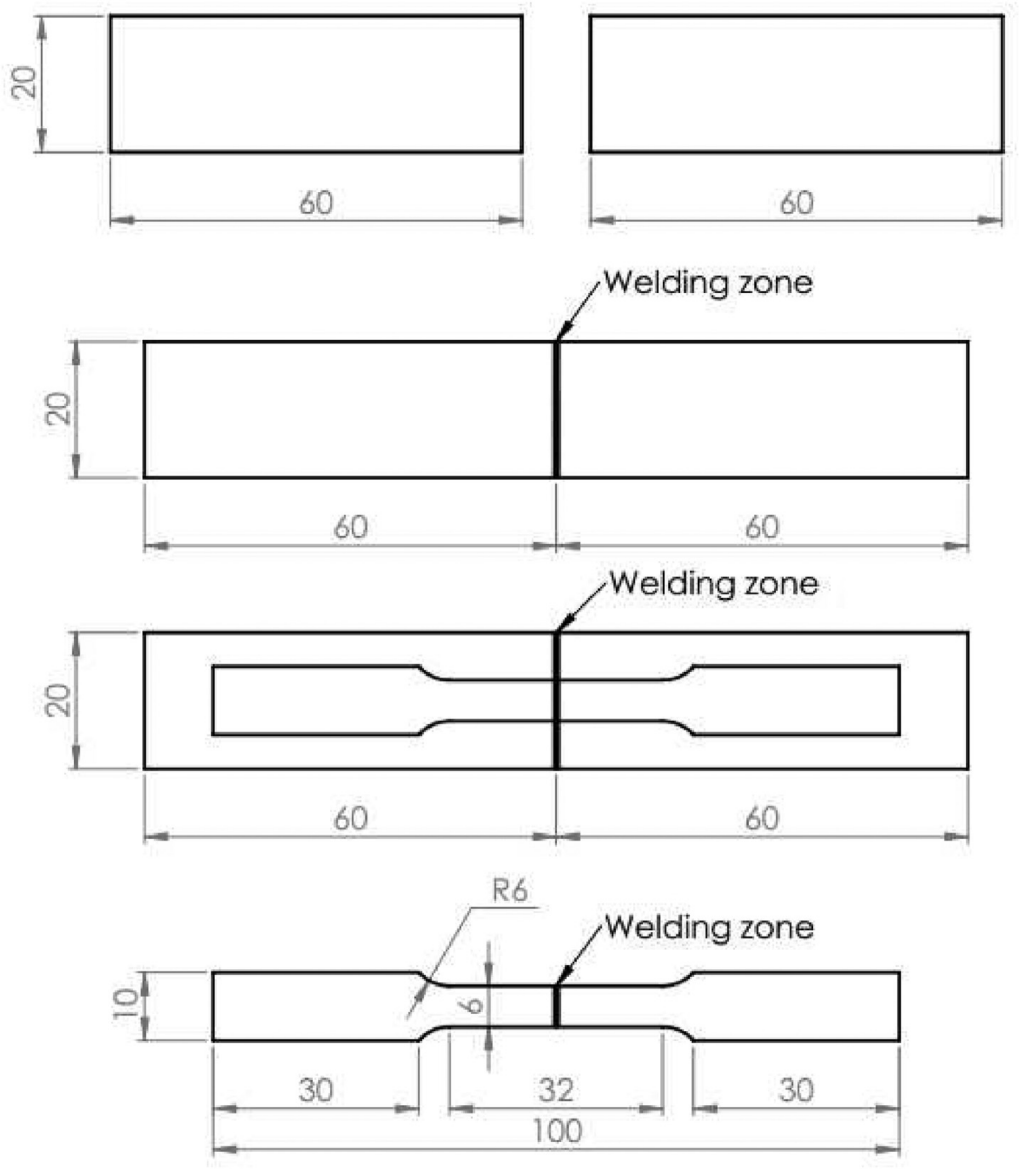
Schematic drawings of the welded sheets and dimensions of the tensile samples with the units shown in millimeters.

The two SPCC steel sheets with 0.5 and 1.0 mm thicknesses were cut from a single SPCC steel sheet via a laser cutting process following the dimension of the welded sheets, which were 60 mm × 20 mm, and used for the laser butt welding. Prior to welding, a silicon carbide sandpaper P600 was used to polish the two sheet pieces and the sample surface was subsequently cleaned with acetone to remove the oxide film. The sheets with the prescribed dimensions were welded first before the tensile samples were laser cut from the welded joints based on the American Society for Testing and Materials (ASTM) E8/E8M standards [17], as shown in Figure 2. Notice that the thicker sheet (20 × 60 × 1 mm^3^) is positioned on the left side of the welding direction while the thinner sheet (20 × 60 × 0.5 mm^3^) is positioned on the right side for butt welding. The workpieces were clamped on a worktable using the strap clamps to prevent any distortion and minimize the residual stress variation between one welding condition to another.

An Einstein Schweissen 530Li Nd:YAG pulsed laser welding machine was employed throughout the study. The welding was carried out in a butt joint configuration without filler metal. The laser head was tilted at an approximate 5° angle from the vertical position to avoid potential damage due to the laser beam back reflection. The weld pool was also protected by a layer of argon shielding gas. The focal plane was located on the workpiece top surface with a focal length of the optics of 110 mm used for the laser delivery. The workpieces to be welded were closely assembled and no gap was reserved purposely.

In order to attain the desirable features from the laser welding process, it is imperative to understand the impact of the laser welding parameters on the weld quality. Numerous studies have explored the varying effects of the welding parameters on the resulting weld geometry. Previously, Duley [18] revealed that the weld seam width corresponded with the welding quality. It was also revealed that the strength of laser-welding joints was affected by the weld geometry since the laser-welded joints were non-axisymmetric [19]. Furthermore, Chen *et al.* [20] employed conventional destructive techniques to measure and investigate the association between the weld seam geometry and the tensile strength. In another study, Li *et al.* [21] indicated that the weld width of the laser welding affected the forming properties of the tailor blanks and led to a greater degree of fracture failure during the stamping process. This is due to the significant effect of the laser welding width on the plasticity of tailor blanks, which can be regulated by optimizing the welding parameters. However, it is practically challenging to directly estimate the width of the bottom weld seam. Therefore, the width of the bottom weld seam was evaluated based on the shape of the top weld seam [22].

Given that multiple weld qualities, including the weld width and weld strength, are controlled by the process parameters, the use of laser beam welding under unsuitable conditions could reduce its effective welding performance. In particular, (1) an excess laser power or (2) an improper pulse shape of the primary laser beam would result in the penetration, deformation, or vaporizing of the welding target. Besides, the (3) beam diameter, (4) flow rate of shielding gas, (5) laser offset, and (6) conveying speed of the workpiece during the welding make up the six crucial welding parameters that were considered in this study, as shown in Table 3.

**Table 3:** The control parameters and levels of the six welding parameters.

Parameters	Symbols	Level 1	Level 2	Level 3
Beam diameter (mm)	A	0.6	0.8	
Flow rate (L/min)	B	3	8	
Power (kW)	C	0.6	1	1.5
Welding speed (mm/s)	D	1	1.6	2.5
Laser offset (mm)	E	0	0.1	0.2
Pulse shape	F	I	II	III

**Figure 3:**
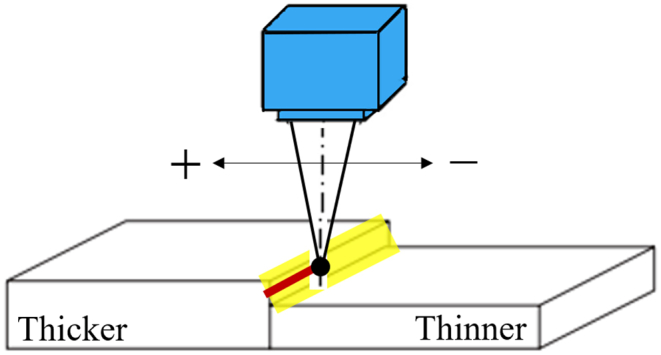
Position at different offsets.
(F) Pulse shape: The applied pulse shape of the laser beam consists of three distinctly-shaped laser pulses that were precisely altered by regulating the panel of the Nd:YAG pulsed laser welding machine, as depicted in Figure 4. Pulse shaping allows the operator to define a laser waveform over multiple segments or points. Programming is accomplished by defining segments in both amplitude (power percentage, %) and duration (time, ms).

The six independently controllable welding parameters for this study are defined as:

(A) Beam diameter: The diameter of the laser beam (mm) measured at the exit face of the laser housing;
(B) Flow rate: The gas flow rate of shielding gas (L/min) at the output of the nozzle measured using a flowmeter;
(C) Laser power: The specific energy (kW) applied by the laser welding device;
(D) Welding speed: The speed (mm/s) at which the workpiece is conveyed along the welding path;
(E) Laser offset: A specific distance (mm) from the interface on the top surface of one of the two materials. When the laser beam is pointed at the top surface of the thicker sheet side, the offset was defined as a positive offset and vice versa (negative offset on the thinner sheet side), as shown in Figure 3;

**Figure 4:**
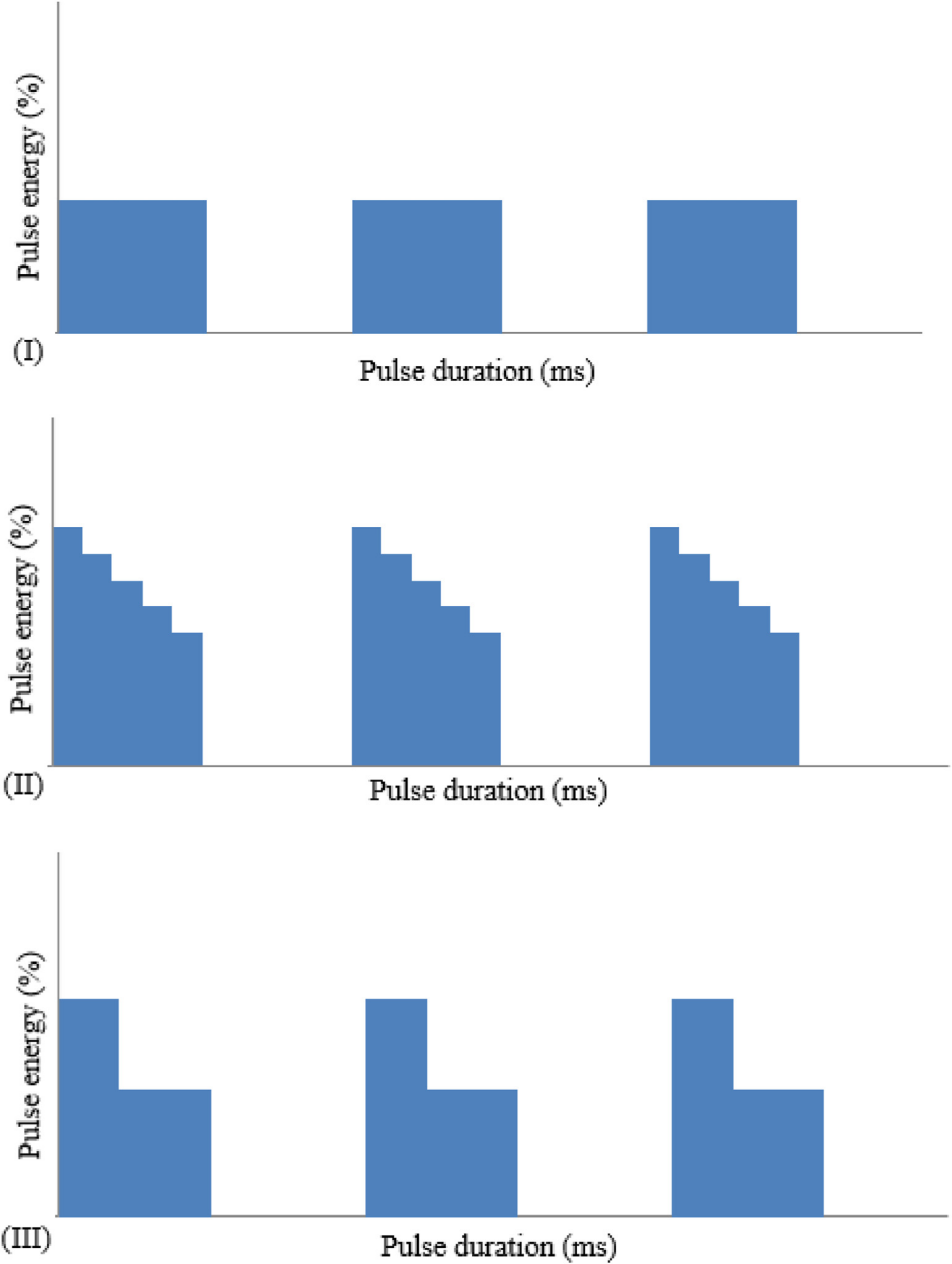
The three distinctly-shaped laser pulses applied in this study.

The classical rectangular pulse shape I, known as the square pulse, was generated via a single rectangular input signal of the laser flash-lamps for the dispersal of heat energy into the workpiece and the surface absorption conditions of the laser radiation. While a square welding pulse is used in most welding cases, few applications have reported an enhanced welding performance using pulse shaping [23]. However, certain laser power supplies are equipped with temporal shaping during each pulse, therefore providing comparably high power densities at the start or the end of the pulse. Such pulse shaping trends have been stated to benefit certain applications, such as to control solidification cracking and cooling rates in crack-sensitive materials [24, 25].

In pulse shape II or the annealing pulse, a few milliseconds were split into five equal length sectors with a 20% decrement of the sector peak power. Pulse shape II can be used to reduce the thermal cycling experienced by the metal sheet when the welding materials are susceptible to cracking [23]. The laser power was then continuously reduced until reaching zero. The average laser energy in pulse shape II decreased as a result of the pulse shaping compared to the laser beam welding in pulse shape I. Hence, welding defects, e.g. cavity shrinkage and porosity, can be reduced and/or avoided [1].

In contrast to pulse shapes I and II, pulse shape III, known as the spike pulse, was composed of a two-sector pulse. During the first sector pulse, a leading-edge spike in the laser pulse surpassed the peak pulse power around 75–100%, while the second sector pulse was set to be 50–60% of the main sector height to ensure the output pulse as rectangular as possible. When the peak power was increased, a considerably deeper weld pool was acquired and was characterized as a penetration or keyhole mode welding. A small molten pool was produced by each laser pulse that resolidifies within a few milliseconds. Conversely, the use of a low peak power caused the welding to take place under the conduction mode, producing a shallow and smooth weld pool [26]. The pulse shape III was used to overcome high reflectivity on materials, such as copper or aluminum [23].

The effect of pulse shapes I, II, and III on the weld qualities was examined. In pulse shape II, the peak power varied within each pulse shape, where the average peak power was equal to the middle sector peak power of each ramped pulse. Based on the applied pulse shapes, the average peak power of pulse shape II was adjusted to correspond to the peak power in pulse shape III. Thus, the average peak power of pulse shape II was similar to the corresponding pulse shape III although the maximum and minimum peak powers of pulse shape II were 140% and 60% with respect to the average peak power, respectively [27].

The dimension of the tensile specimens cut from the welded sheets is shown in Figure 2. The tensile test was conducted at room temperature and a crosshead speed of 10 mm/min with the weld positioned at the center of the gauge length. The prepared sample was clamped by the testing machine jaws and subjected to a gradually increased tensile force via the mechanical lever system until the sample fractured.

The strength of the weld was determined from the fracture position. Two fracture modes were examined for the butt joints, which substantially influence the maximum attainable tensile force. In the so-called mode I, the fracture occurred at the thinner sheet when the strength of the weld metal surpass that of the base metal. A huge portion of the plastic strain occurred in the base metal with the resultant necking (local reduction in the cross-section area by stretching) and failure emerged outside of the area. In addition, the fracture occurred in the weld on the thinner sheet side invariably for different thickness joints. This condition also occurred due to the increased weld strength, where the fatigue deformation concentrates on the thinner sheet side of the base metal with lower strength and hardness. Therefore, a significant stress concentration occurred in the stepped weld area, leading to fracture in this region. This assumption also justifies the lower fatigue strength of differential thickness joints than equal thickness joints [28].

On the contrary, in the so-called mode II, the fracture occurred when the weld strength was relatively smaller than that of the base metal with a greater share of the plastic strain occurring in the weld and the fracture at the weld seam. Notice that mode I resulted in the most ductile joint fracture while mode II produced the most brittle joint fracture. When a mode II fracture occurred, the fracture surface took place directly at the butt of the joint. It was evident that the maximum tensile strength bearable by the butt joint depends remarkably on the occurring fracture mode, where the tensile strength at fracture mode I was more than mode II.

## 3 Optimization and experimental design

### 3.1 Taguchi design of experiment

The experimental design in this study was based on the Taguchi method to determine the ranking of importance of various process parameters on the target responses. As listed in Table 3, the six process parameters include the beam diameter, laser power, flow rate, welding speed, laser offset, and pulse shape with their respective two or three different levels. Hence, the study considered 324 (2 × 2 × 3 × 3 × 3 × 3) different combinations in total. Nevertheless, the Taguchi method only allows the samples to be arranged into 36 groups yet still achieve a yield result with equivalent confidence if they were examined separately.

The Taguchi method was employed in the laser beam welding process to simultaneously maximize the weld strength and minimize the weld width. Table 4 shows the Taguchi-based L36 (2ˆ2 3ˆ4) orthogonal array along with the quality characteristics. The experiment number indicates the diverse experimental levels of the various factors. The weld strength was assessed using a Cometech QC-506M1 universal testing machine and calculated as the Ultimate Tensile Strength (UTS) per unit length of the weld. In addition, each welding sample was viewed via a Nikon SMZ25 Stereomicroscope with a 0.005 mm accuracy to measure the weld width (Wd) at the center of the seam length. The SNRs for a given response and the predicted SNRs of the starting conditions were calculated using Eq. (1) or (2) depending on the type of quality characteristics.

**Table 4:** The response value and SNRs of the process parameters based on the experimental numbers.

Exp no.	Process parameter level						Responses		SNR for responses	
	*A*	*B*	*C*	*D*	*E*	*F*	UTS (Mpa)	Wd (mm)	UTS	Wd
1	1	1	1	1	1	1	358	0.76	50.6079	2.21234
2	1	1	2	2	2	2	222	0.82	46.4018	1.72372
3	1	1	3	3	3	3	248	1.10	47.5697	-0.15967
4	1	1	1	1	1	1	323	0.79	∗	∗
5	1	1	2	2	2	2	198	0.82	∗	∗
6	1	1	3	3	3	3	231	0.93	∗	∗
7	1	1	1	1	2	3	129	0.85	42.2118	1.41162
8	1	1	2	2	3	1	200	0.74	46.0206	2.61537
9	1	1	3	3	1	2	155	1.10	43.8066	-0.82785
10	1	2	1	1	3	2	187	0.80	45.4368	1.93820
11	1	2	2	2	1	3	208	0.89	46.3613	1.01220
12	1	2	3	3	2	1	215	1.02	46.6488	-0.17200
13	1	2	1	2	3	1	301	1.10	49.5713	-0.82785
14	1	2	2	3	1	2	148	1.00	43.4052	0.00000
15	1	2	3	1	2	3	200	0.90	46.0206	0.91515
16	1	2	1	2	3	2	171	1.00	44.6599	0.00000
17	1	2	2	3	1	3	200	0.87	46.0206	1.20961
18	1	2	3	1	2	1	178	0.85	45.0084	1.41162
19	2	1	1	2	1	3	236	1.13	47.4582	-1.06157
20	2	1	2	3	2	1	196	0.88	45.8451	1.11035
21	2	1	3	1	3	2	180	0.85	45.1055	1.41162
22	2	1	1	2	2	3	232	0.99	47.3098	0.08730
23	2	1	2	3	3	1	312	0.80	49.8831	1.93820
24	2	1	3	1	1	2	93	0.86	39.3697	1.31003
25	2	1	1	3	2	1	210	1.01	46.4444	-0.08643
26	2	1	2	1	3	2	246	0.68	47.8187	3.34982
27	2	1	3	2	1	3	18	0.83	25.1055	1.61844
28	2	2	1	3	2	2	274	1.06	48.7550	-0.50612
29	2	2	2	1	3	3	223	0.72	46.9661	2.85335
30	2	2	3	2	1	1	229	0.92	47.1967	0.72424
31	2	2	1	3	3	3	329	1.02	50.3439	-0.17200
32	2	2	2	1	1	1	165	0.79	44.3497	2.04746
33	2	2	3	2	2	2	185	0.94	45.3434	0.53744
34	2	2	1	3	1	2	219	1.13	46.8089	-1.06157
35	2	2	2	1	2	3	194	0.81	45.7560	1.83030
36	2	2	3	2	3	1	265	0.96	48.4649	0.35458

As shown in Table 4, the columns of the arrays are balanced and orthogonal. This means that in each pair of columns, all factor combinations occur the same number of times. Orthogonal designs can be used to estimate the effect of each factor on the response independently of all other factors. Because experiment number 4 to 6 are repeated, the repeated runs show an asterisk instead of doubling the value of the mean and SNR. In other words, the results were printed in that combination once.

The determination of the weld quality was based on the following procedure. The narrow weld zone width in the laser welding enhanced the corrosion resistance of the weld zone [29]. The weld strength of the weld metal exceeded that of the base metal, which was composed of the 258 and 286 MPa class steel sheets with 0.5 and 1.0 mm thicknesses, respectively (Table 2). The weld strength of the welded sample in experiments number 28 and 36 was between the two base material strengths. In contrast, the weld strength was greater than both base metals in experiment number 1, 4, 13, 23, and 31. Whereas in experiments number 13 and 31, the fracture occurred in the thinner/weaker material but not in the weld. The result suggests that the weld region in the two experiments is relatively stronger than those of other regions of the weldment. Regardless of the experimental number, all fractures occurred at the weld seam during the tensile test.

Generally, a higher weld strength during the laser beam welding process enhances the welding performance. Conversely, a lower weld width is also deemed an essential parameter to achieve a decent welding performance. Therefore, this study selected both the weld strength and weld width at different values (the larger-the-better type (L-type) for weld strength and the smaller-the-better type (S-type) for weld width) as the major quality characteristics for simultaneous optimization.

The Taguchi-based L36 orthogonal array method was selected to analyze the input parameters by reducing the number of experiments [2]. The variation between the experimental and desired values was described and measured as the loss function, which was subsequently converted into an SNR that expresses the scatter around the desired value. The ‘signal’ and ‘noise’ represent the desirable and undesirable values, respectively.

In the present study, the selected L-type weld strength and S-type weld width values were used to calculate the SNR for the corresponding responses using the following expression. Primarily, the SNR weld strength for the L-type quality characteristic is determined as:

(1) $$SNR=-10\;\log\left(\frac{1}{n}\right)\sum_{i=1}^{n}\frac{1}{y_{i}^{2}}$$

For the SNR weld width, the S-type quality characteristic is expressed as follows:

(2) $$SNR=-10\;\log\left(\frac{1}{n}\sum_{i=1}^{n}y_{i}^{2}\right)$$

where n is defined as the number of replications for each experiment and $y_{i}$ represents the $i^{th}$ quality characteristics value.

### 3.2 Analysis of Variance (ANOVA)

The Minitab 19 statistical software was employed for the Analysis of Variance (ANOVA) and Taguchi analysis. The ANOVA is a useful statistical technique to determine the individual interactions as well as the contribution ratio of all parameters in the experimental setup. The effect of the six process parameters (beam diameter, laser power, flow rate, welding speed, laser offset, and pulse shape) on the responses (weld strength and weld width) was determined using the ANOVA. Subsequently, the outcome of the ANOVA, which indicates the importance order of the influential parameters on the response, was assessed to confirm the obtained results through the Taguchi method.

### 3.3 Confirmation experiment

In reference to Krishnaiah and Shahabudeen [30], Taguchi recommended a confirmation experiment as a convenient method to verify the model with the selected factors set at optimum levels. The additive model is considered valid if the confirmation result is comparable to the predicted response based on the main effects model. Thereby, the model can be effectively used to estimate the response for any combination of the factor levels within the experimentation region.

The optimum condition can be predicted by calculating the expected mean at the optimal settings (μ) using Eq. (3).

(3) $$\mu =\gamma_{m}+\sum_{i=1}^{n}\left({\bar{\gamma }}_{i}-\gamma_{m}\right)$$

where $\gamma_{m}$ represents the grand average, ${\bar{\gamma }}_{i}$ refers to the mean value of $i^{th}$ parameter at an optimum level, and n is defined as the number of controllable variables that substantially affect the quality characteristic.

The Confidence Interval (CI) for the confirmation experiment is given by:

(4) $$CI=\sqrt{F_{\alpha ,v_{1},v_{2}}MS_{e}\left[\frac{1}{n_{eff}}+\frac{1}{r}\right]}$$

where $F_{\alpha ,v_{1},v_{2}}$ is the value found in the F-distribution table with $v_{1}$ (the degree of freedom, often abbreviated df, of the numerator related to the mean, which is fixed to 1), $v_{2}$ (the degree of freedom of the mean squared error), and a significance level of α. $MS_{e}$ is the mean squared error, and r is the sample size used in the confirmation experiment.

Additionally, the effective number of observations ($n_{eff}$) is given by Eq. (5).

(5) $$n_{eff}=\frac{Total\;number\;of\;observations}{1+total\;df\;of\;effects\;used\;in\;\mu }$$

Therefore, a CI range of 95% of the confirmation experiment is expressed as:

(6) $$\mu -CI\leq \mu_{conf}\leq \mu +CI$$

where $\mu_{conf}$ is the mean value after the confirmation experiment was performed under the optimal setting point.

### 3.4 CRITIC method

Depending on the calculated weights of criteria using CRITIC [11], the weight of the $j^{th}$ response,$w_{j}$ was carried out by characterizing each vector based on the Standard Deviation (SD), followed by the construction of a symmetric matrix with the linear correlation coefficients between the vectors [31].

First, an initial decision matrix $D={\left[d_{ij}\right]}_{m\times n}$ that consists of m quality characteristics and n criteria is defined. Term $d_{ij}$ depicts the output value of $i^{th}$ alternative with respect to $j^{th}$ criterion. The defined *D* was normalized to avoid numerical fluctuations of various quality characteristics with output values between 0 and 1, and can be expressed as:

(7) $${\bar{d}}_{ij}=\frac{d_{ij}-d_{j}^{worst}}{d_{j}^{best}-d_{j}^{worst}}$$

where ${\bar{d}}_{ij}$ represents the normalized output value of $i^{th}$ alternative for $j^{th}$ criterion, while $d_{j}^{worst}$ and $d_{j}^{best}$ depict the least and best values of the $j^{th}$ criterion output, respectively. In addition, $d_{ij}$ is expressed with the output value of $i^{th}$ alternative associated with $j^{th}$ criteria, where i=1,2,...,m;j=1,2,...,n.

Subsequently, the intensity of the criteria contrast was determined according to the SD of the normalized criterion values by columns ($d_{j}$). The SD of the $j^{th}$ criterion, $\sigma_{j}$ is expressed as:

(8) $$\sigma_{j}=\sqrt{\frac{\sum_{i=1}^{m}{\left({\bar{d}}_{ij}-{\bar{d}}_{j}\right)}^{2}}{m}}$$

where m implies the number of experiments and ${\bar{d}}_{j}$ is defined as the average output values of the $j^{th}$ criterion.

Next, the symmetrical matrix (m×m) was established with the linear correlation coefficient between the criteria, $r_{jk}$ can be expressed as:

(9) $$r_{jk}=\frac{\sum_{i=1}^{m}\left({\bar{d}}_{ij}-{\bar{d}}_{j}\right)\left({\bar{d}}_{ik}-{\bar{d}}_{k}\right)}{\sum_{i=1}^{m}{\left({\bar{d}}_{ij}-{\bar{d}}_{j}\right)}^{2}\sum_{i=1}^{m}{\left({\bar{d}}_{ik}-{\bar{d}}_{k}\right)}^{2}}$$

The correlation coefficient is a statistical measure that varies from -1 to 1 to indicate the strength of the relationship between the two criteria. A positive coefficient value reflects the increase or decrease of the criteria together while a negative coefficient value reflects the inverse relationship between the criteria.

After that, the criterion information contained in the $j^{th}$ criterion, $c_{j}$ was calculated by multiplicative formulae of Eqs. (8) and (9) as:

(10) $$c_{j}=\sigma_{j}\sum_{k=1}^{m}\left(1-r_{jk}\right)$$

According to Eq. (10), the larger the value $c_{j}$ is, the greater the amount of information transmitted by the corresponding criterion. Therefore, the relative importance and the objective weight of the criteria are also greater [11].

Finally, the objective weight attributed to the $j^{th}$ criteria, $w_{j}$, was determined by applying the normalized technique with the help of the obtained criterion information, as expressed in the following:

(11) $$w_{j}=\frac{c_{j}}{\sum_{j=1}^{n}c_{j}}$$

### 3.5 Grey Relational Analysis (GRA)

The Taguchi method is technically inappropriate to conduct simultaneous multi-objective optimization. Therefore, the GRA method was utilized as an alternative approach to determine the ranking of importance of each process parameter on the multiple quality characteristics by simultaneously maximizing the weld strength as well as minimizing the weld width. Usually, GRA considers the dimension of factors that exhibit a large magnitude difference. Thus, the magnitude of the original data is normalized to one and dimensionless [8].

Primarily, the original response data was converted into the SNR ($y_{ij}$) depending on the type of quality characteristic. Then, the $y_{ij}$ was normalized as $x_{ij}$ into the range [0,1], which is termed as the grey relational generating. Since the normalization process affects the ranking, the sensitivity of the normalization process on the sequencing results was also analyzed [30].

The normalized results, $x_{ij}$ for the L-type quality characteristic of the weld strength is expressed as:

(12) $$x_{ij}=\frac{y_{ij}-\min\left(y_{ij}\right)}{\max\left(y_{ij}\right)-\min\left(y_{ij}\right)}$$

Conversely, the normalized results, $x_{ij}$ for the S-type quality characteristic of the weld width is expressed as:

(13) $$x_{ij}=\frac{\max\left(y_{ij}\right)-y_{ij}}{\max\left(y_{ij}\right)-\min\left(y_{ij}\right)}$$

A larger normalized result refers to a better weld quality, where an ideal normalized result should be equal to 1. For a $j^{th}$ response of an $i^{th}$ experiment, the performance of $i^{th}$ experiment is considered the best if the value $x_{ij}$, which has been normalized, is equal to or close to 1 than the value of other experiments. The reference sequence, $X_{0}$ is expressed as $\left(x_{01},x_{02},...,x_{0j},...,x_{0n}\right)=\left(1,1,...,1,...,1\right)$, where $x_{0j}$ refers to the reference value for the $j^{th}$ response and is used to identify the experiment with the closest comparability to the reference sequence.

Following the normalization, the Grey Relational Coefficient (GRC) was used to determine the range between $x_{ij}$ and $x_{0j}$, where a larger GRC indicates a closer value between the two. The GRC for the normalized SNR values is expressed as:

(14) $$\begin{array}{cccc} \gamma \left(x_{0j},x_{ij}\right)=\frac{\left(\Delta_{\min}+\xi \;\Delta_{\max}\right)}{\left(\Delta_{ij}+\xi \;\Delta_{\max}\right)} & for & i=1,2,...,m & \begin{array}{cc} and & j=1,2,...,n \end{array} \end{array}$$

where

1. $\gamma \left(x_{0j},x_{ij}\right)$ is the GRC between $x_{ij}$ and $x_{0j}$
2. $\Delta_{ij}=\left|x_{0j}-x_{ij}\right|$ indicates the absolute value of the difference between $x_{ij}$ and $x_{0j}$
3. $\Delta_{\min}=\min\left\{\Delta_{ij},i=1,2,...,m;j=1,2,...,n\right\}$ is the smallest value of $x_{ij}$
4. $\Delta_{\max}=\max\left\{\Delta_{ij},i=1,2,...,m;j=1,2,...,n\right\}$ is the largest value of $x_{ij}$
5. ξ is the distinguishing coefficient. A high distinguishability is represented by a small ξ. The ξ is used to reduce the impact of $\Delta_{\max}$ when its value becomes too large and thus increase the significant difference of the GRC. The ξ is fixed to 0.5 if all the process parameters exhibit equal weighting [12].

The quantification of the grey relational space is known as the Grey Relational Grade (GRG), which is the weighted sum of the GRCs, and is determined using the following:

(15) $$\Gamma \left(X_{0},X_{i}\right)=\sum_{j=1}^{n}w_{j}\gamma \left(x_{0j},x_{ij}\right)\begin{array}{ccc} & for & i=1,2,...,m \end{array}$$

where $\sum_{j=1}^{n}w_{j}=1$

The GRG, $\Gamma \left(X_{0},X_{i}\right)$ corresponds to the degree of similarity between the comparability sequence, $X_{i}$ and the reference sequence, $X_{0}$. An experiment that records the highest GRG implies that the comparability sequence is almost similar to the reference sequence, thus, making that experiment the preferred option. The response graph method or ANOVA was used and the optimal levels of the factors were selected based on the maximum average $\Gamma \left(X_{0},X_{i}\right)$ value.

Eventually, the obtained optimum condition for the multiple quality characteristics was predicted and verified via the confirmation experiment once the optimum combined process parameters were selected. The predicted optimum GRG, $\hat{\gamma }$ was calculated based on that of Haq *et al.* [13]:

(16) $$\hat{\gamma }=\gamma_{t}+\sum_{i=1}^{n}\left(\gamma_{i}-\gamma_{t}\right)$$

where $\gamma_{t}$ is defined as the total mean of the GRG, $\gamma_{i}$ refers to the mean GRG of $i^{th}$ parameter at optimum levels, and n is the number of controllable variables that significantly influenced the quality characteristics.

The Confidence Interval (CI) for the confirmation experiment was calculated using Eq. (4), while the effective number of observations ($n_{eff}$) is expressed as Eq. (17).

(17) $$n_{eff}=\frac{Total\;number\;of\;observations}{1+total\;df\;of\;effects\;used\;in\;\hat{\gamma }}$$

Therefore, a 95% CI range of the predicted optimum condition is determined as:

(18) $$\hat{\gamma }-CI\leq {\hat{\gamma }}_{cgg}\leq \hat{\gamma }+CI$$

where ${\hat{\gamma }}_{cgg}$ is the GRG value after the confirmation experiment was performed under the optimal setting point.

## 4 Results and discussion

### 4.1 Signal-to-Noise Ratio (SNR) analysis

#### 4.1.1 Weld strength response analysis

**Figure 5:**
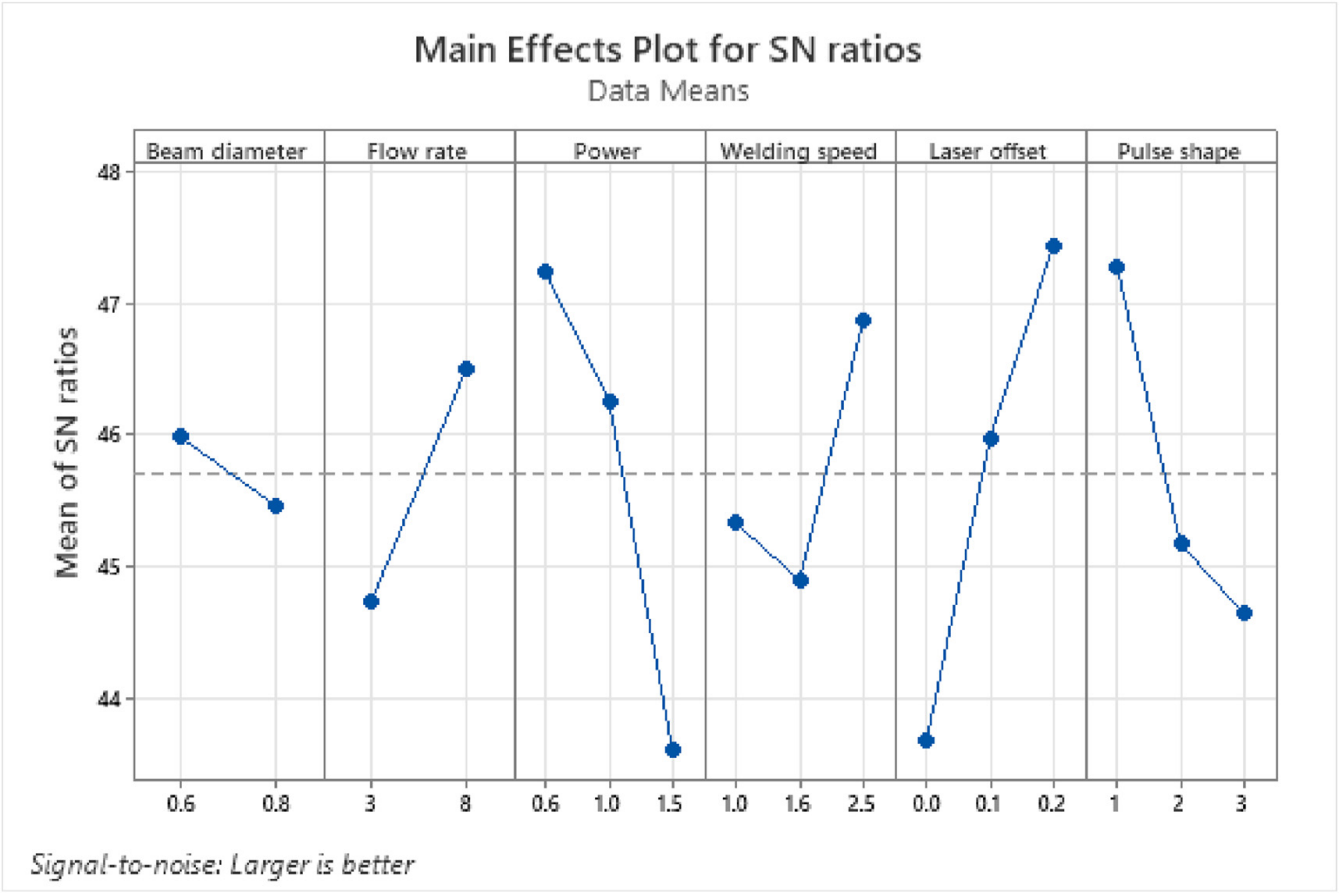
The main effect plots of the SNRs on the weld strength.

Table 5 describes the average SNRs and the ranking of importance of the process parameters of the weld strength, while Figure 5 illustrates the primary effect of each parameter on the weld strength of the laser-welded samples. Based on Figure 5, the maximum weld strength was obtained using the optimum process parameters A_1_B_2_C_1_D_3_E_3_F_1_, as follows: beam diameter = 0.6 mm, flow rate = 8 L/min, power = 0.6 kW, welding speed = 2.5 mm/s, laser offset = 0.2 mm, and pulse shape = I (Figure 5). Canbolat *et al.* [32] stated that the effect of the process parameter on the response is relatively low when the lowest and highest SNRs variation is small. Moreover, the maximum SNRs of the design parameters indicate the optimum condition of the system. Therefore, the optimum weld strength was achieved when the laser welding process was performed under optimum working conditions of A_1_B_2_C_1_D_3_E_3_F_1_. The result correlated with the study by Cao *et al.* [33] who reported that an increase in the laser offset yielded an initial increment of the tensile strength before the value sharply decreased. The maximum value of the laser offset was also 0.2 mm.

**Table 5:** Response table for the SNRs on the weld strength.

Level	Beam diameter	Flow rate	Power	Speed	Laser offset	Pulse shape
1	**45.98**	44.73	**47.24**	45.33	43.68	**47.28**
2	45.46	**46.51**	46.26	44.90	45.98	45.17
3			43.60	**46.87**	**47.44**	44.65
Delta	0.52	1.78	3.63	1.97	3.76	2.63
Rank	6	5	2	4	1	3

Bold value signifies to show the level at which the optimum condition is achieved for each process parameter.

The ANOVA was applied to verify the Taguchi analysis and ensure that the results were statistically reliable [30]. The percentage contribution of each parameter of the laser-welded samples on the weld strength was estimated under different conditions. As described in Table 6, the ANOVA result shows the influence of the process parameters on the weld strength.

**Table 6:** ANOVA result of the effect of process parameters on the weld strength.

Source	DF	Seq SS	Contribution	Adj SS	Adj MS	F-Value	P-Value
Beam diameter	1	121	0.08%	121.0	121.0	0.03	0.853
Flow rate	1	300	0.20%	300.4	300.4	0.09	0.771
Power	2	25113	16.64%	25112.7	12556.4	3.62	0.042∗
Speed	2	3951	2.62%	3950.7	1975.4	0.57	0.573
Laser offset	2	14190	9.40%	14190.1	7095.0	2.05	0.150
Pulse shape	2	20478	13.57%	20477.6	10238.8	2.95	0.071
Error	25	86729	57.48%	86728.7	3469.1		
Total	35	150881	100.00%				

∗Significant at 5% level.

Based on the ANOVA results, the laser power was the major process parameter that affected the weld strength of the laser-welded samples with an impact ratio of 16.64%. The overall contribution ratios in Table 6 indicate that the importance ranking of the process parameters on the weld strength was in the order of power > pulse shape > laser offset > welding speed > flow rate > beam diameter. Interestingly, both the primary effect plots of the Taguchi method and the ANOVA results recorded a comparable ranking of the process parameter effect on the weld strength of the laser-welded samples. The results in the present study correlated with the findings on the effect of laser power on the weld strength by Akman *et al.* [34], where the laser power increment had a direct influence on the decreased weld strength. Dieter [35] also reported a similar finding in which the increased laser power generally tends to increase the grain size of the laser beam welding, thus, demonstrating the tendency of the mechanical properties to decrease in the laser-welded samples. Therefore, an increment in the laser power of the laser-welded sample compared to the other process parameters resulted in the largest impact on the weld strength.

#### 4.1.2 Weld width target function analysis

**Figure 6:**
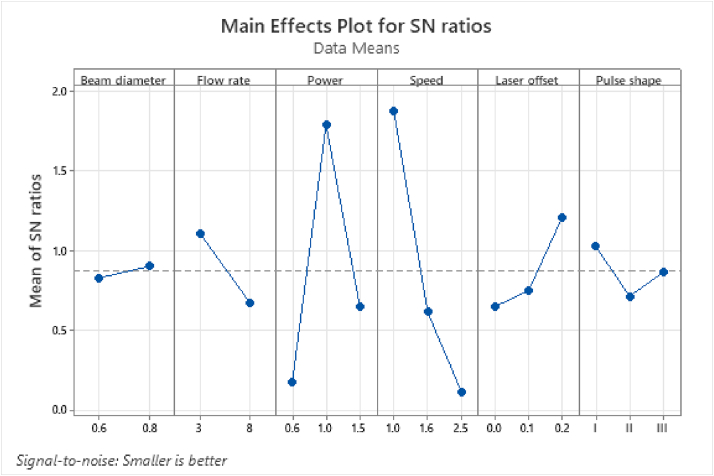
The main effect of the SNR of each parameter on weld width.

Table 7 provides the response of the weld width based on the average SNRs and the rank of importance of the process parameters, while Figure 6 illustrates the effect of each parameter on the weld width. From Table 7, the welding speed was ranked first among the six process parameters with a higher influence on the weld width of the laser-welded sample. The optimum process parameters A_2_B_1_C_2_D_1_E_3_F_1_, which corresponds to the beam diameter = 0.8 mm, flow rate = 3 L/min, power = 1 kW, welding speed = 1 mm/s, laser offset = 0.2 mm, and pulse shape = I, achieved the minimum weld width, as shown in Figure 6. The findings corroborate with the study by Vyskoč *et al.* [36] who stated that the weld width slightly decreased with a reduced gas flow rate.

**Table 7:** Response table for the SNRs on the weld width.

Level	Beam diameter	Flow rate	Power	Speed	Laser offset	Pulse shape
1	0.8308	**1.1102**	0.1758	**1.8810**	0.6530	**1.0298**
2	**0.9047**	0.6719	**1.7900**	0.6167	0.7512	0.7159
3			0.6476	0.1157	**1.2092**	0.8677
Delta	0.0739	0.4383	1.6142	1.7654	0.5562	0.3139
Rank	6	4	2	1	3	5

Bold value signifies to show the level at which the optimum condition is achieved for each process parameter.

Additionally, the ANOVA result in Table 8 shows that the welding speed and laser power demonstrated the most significant effect on the weld width of the laser-welded samples. According to the contribution ratio, the important order of the process parameters on the weld width of the laser-welded samples was in the order of welding speed > power > flow rate > pulse shape > laser offset > beam diameter, where the highest contribution ratio of the welding speed was up to 41.39%. In contrast, the beam diameter was less influential on both the target functions (weld strength and weld width) of the laser-welded samples compared to other parameters. The result is in agreement with that of Li *et al.* [21], which revealed the laser power and welding speed as the two main influential parameters on the weld geometry, including its depth and width. Given that the laser power corresponds with the width of the weld seam, thus, the increase of the laser power also increased the width of the weld seam up to a certain limit [37].

**Table 8:** The effect of process parameters on the weld width based on the ANOVA results.

Source	DF	Seq SS	Contribution	Adj SS	Adj MS	F-Value	P-Value
Beam diameter	1	0.000044	0.01%	0.000044	0.000044	0.01	0.925
Flow rate	1	0.019600	3.69%	0.019600	0.019600	4.03	0.055
Power	2	0.153622	28.95%	0.153622	0.076811	15.81	0.000∗
Speed	2	0.219622	41.39%	0.219622	0.109811	22.61	0.000∗
Laser offset	2	0.005939	1.12%	0.005939	0.002969	0.61	0.551
Pulse shape	2	0.010289	1.94%	0.010289	0.005144	1.06	0.362
Error	25	0.121439	22.89%	0.121439	0.004858		
Total	35	0.530556	100.00%				
∗Significant at 5% level							

#### 4.1.3 Confirmation test

The confirmatory test was performed to accurately assess the appropriate parameters for the optimization process. As shown in Table 9, the computed $\mu_{conf}$ values using Eq. (6) indicate that the optimum experimental values for each process parameter were within an acceptable range.

**Table 9:** Optimum experimental values of each target function under optimum conditions.

Target Function	Optimum condition	Optimum value by Experiment	$\mu_{conf}$
Weld strength (MPa)	A_1_B_2_C_1_D_3_E_3_F_1_	320	121 $\leq \mu_{conf}\leq$ 373
Weld width (mm)	A_2_B_1_C_2_D_1_E_3_F_1_	0.66	0.56 $\leq \mu_{conf}\leq$ 0.87

### 4.2 CRITIC method

The CRITIC method was employed to determine the objective criteria weights based on the experimental data by eliminating the decision makers’ effect on the decision-making process in order to achieve optimal conditions for both the weld strength and weld width. Following the Taguchi-based orthogonal array approach as an alternative for the decision-making process, the output values for all 36 experimental trials were normalized between 0 and 1 using Eq. (7). Table 10 presents the normalized values and the SD values corresponding to each output using Eq. (8).

**Table 10:** Normalized values of the responses based on the CRITIC method.

Experiment no.	Responses	
	Weld strength	Weld width
1	1.0000	0.8161
2	0.6005	0.6782
3	0.6763	0.0575
4	0.8973	0.7586
5	0.5296	0.6897
6	0.6267	0.4368
7	0.3253	0.6207
8	0.5351	0.8506
9	0.4026	0.0575
10	0.4987	0.7241
11	0.5583	0.5287
12	0.5784	0.2414
13	0.8335	0.0690
14	0.3835	0.2874
15	0.5369	0.4943
16	0.4495	0.2759
17	0.5355	0.5747
18	0.4721	0.6092
19	0.6419	0.0000
20	0.5245	0.5402
21	0.4779	0.6092
22	0.6304	0.2989
23	0.8662	0.7241
24	0.2205	0.5862
25	0.5655	0.2529
26	0.6710	1.0000
27	0.0000	0.6552
28	0.7520	0.1379
29	0.6035	0.8966
30	0.6202	0.4483
31	0.9151	0.2299
32	0.4336	0.7586
33	0.4914	0.4138
34	0.5900	0.0000
35	0.5168	0.7126
36	0.7263	0.3793
SD	0.1932	0.2736

The results of the estimated correlation coefficient and the weights of individual outputs are shown in Table 11. Eq. (9) was used to evaluate the correlation coefficient of each criterion and obtain the symmetrical matrix (m×m) values minus one from Table 10, while Eqs. (10) and (11) was applied to calculate the weights of the individual response following the criterion information. The weights corresponding to the weld strength and weld width were equal to 0.4157 and 0.5843, respectively. The identified weights show a good representation of specific requirements in the LWBs, where weld width in a blank has prime importance as it greatly influences the formability and corrosion behavior [21, 29]. Also, weld strength is essential as it directly affects the quality measures and fracture position of the welded sample [28].

**Table 11:** The correlation coefficient of each criterion and objective weights of both responses.

Criteria	Correlation coefficient		Objective weights	
	Weld strength	Weld width	$c_{j}$	$w_{j}$
Weld strength	1	-0.0829	0.2107	0.4157
Weld width	-0.0829	1	0.2963	0.5843

### 4.3 Grey Relational Analysis (GRA)

The CRITIC method was also utilized to determine the weight fraction of each response. The GRA-based Taguchi method was performed to achieve the optimal parametric combination of high weld strength and low weld width in the laser beam welding.

The SNR values were initially normalized according to Eqs. (12) and (13), followed by Eq. (14) to calculate the GRC. The GRG was then obtained by converting the multiple quality characteristics into a single performance characteristic using the GRCs with CRITIC weighting via Eq. (15). The GRG was estimated based on GRG=0.4157UTS+0.5843Wd before the final ranking was determined. Finally, the grades were considered for the multi-criteria optimization problem. Table 12 presents the normalized results, GRCs, GRG, and rank for each experimental number.

**Table 12:** Normalized results, GRC, GRG, and rank for each experimental number.

Exp. No.	Normalized Results		Grey Relational Coefficients		GRG	Rank
	Weld strength	Weld width	Weld strength	Weld width		
1	1.0000	0.2579	1.0000	0.4025	0.6535	14
2	0.8351	0.3686	0.7520	0.4419	0.5721	22
3	0.8809	0.7956	0.8076	0.7098	0.7509	7
4	∗	∗	∗	∗	∗	∗
5	∗	∗	∗	∗	∗	∗
6	∗	∗	∗	∗	∗	∗
7	0.6708	0.4394	0.6030	0.4714	0.5267	29
8	0.8201	0.1665	0.7354	0.3750	0.5264	30
9	0.7333	0.9470	0.6522	0.9042	0.7983	6
10	0.7972	0.3200	0.7115	0.4237	0.5446	26
11	0.8335	0.5299	0.7502	0.5154	0.6140	19
12	0.8448	0.7983	0.7631	0.7126	0.7338	8
13	0.9594	0.9470	0.9248	0.9042	0.9129	2
14	0.7176	0.7594	0.6390	0.6751	0.6599	13
15	0.8201	0.5519	0.7354	0.5274	0.6148	18
16	0.7668	0.7594	0.6819	0.6751	0.6780	12
17	0.8201	0.4852	0.7354	0.4927	0.5946	21
18	0.7804	0.4394	0.6949	0.4714	0.5653	24
19	0.8765	1.0000	0.8019	1.0000	0.9168	1
20	0.8132	0.5077	0.7281	0.5039	0.5980	20
21	0.7842	0.4394	0.6986	0.4714	0.5668	23
22	0.8707	0.7396	0.7945	0.6575	0.7151	10
23	0.9716	0.3200	0.9462	0.4237	0.6432	16
24	0.5593	0.4624	0.5315	0.4819	0.5027	32
25	0.8367	0.7789	0.7539	0.6934	0.7188	9
26	0.8906	0.0000	0.8205	0.3333	0.5380	27
27	0.0000	0.3925	0.3333	0.4515	0.4018	33
28	0.9273	0.8741	0.8731	0.7988	0.8300	4
29	0.8572	0.1125	0.7778	0.3604	0.5357	28
30	0.8662	0.5952	0.7889	0.5526	0.6519	15
31	0.9896	0.7983	0.9797	0.7126	0.8248	5
32	0.7546	0.2952	0.6708	0.4150	0.5224	31
33	0.7936	0.6375	0.7078	0.5797	0.6335	17
34	0.8510	1.0000	0.7705	1.0000	0.9036	3
35	0.8097	0.3445	0.7244	0.4327	0.5552	25
36	0.9160	0.6790	0.8561	0.6090	0.7128	11

Krishnaiah and Shahabudeen [30] stated that the optimum combination of process parameters is indicated by the experiment with the maximum GRG among all the experiments. Based on this statement, experiment number 19 achieved the highest GRG and was therefore considered the best multi-response characteristics among the 36 experiments. The optimum controllable parameters combination corresponds to the beam diameter = 0.8 mm (level 2), flow rate = 3 L/min (level 1), power = 0.6 kW (level 1), welding speed = 1.6 mm/s (level 2), laser offset = 0 mm (level 1), and pulse shape = type III (level 3).

**Figure 7:**
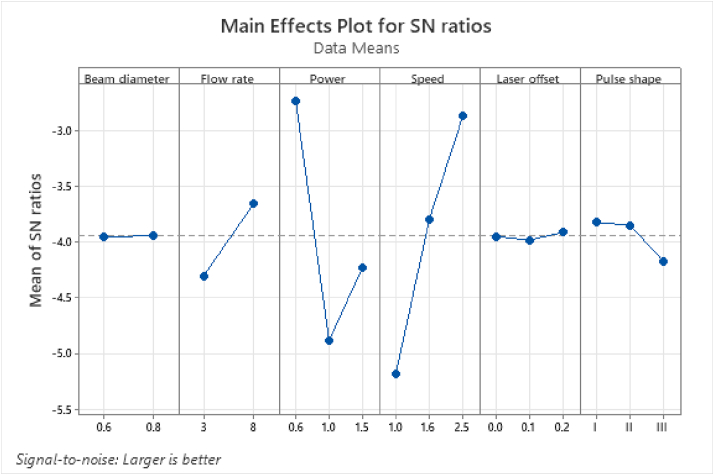
The SNR plot of the overall GRG.

Table 13 shows the mean response table of the overall GRG, which was calculated using Eq. (15), while Figure 7 presents the SNRs of the overall GRG calculated using the L-type quality characteristic. According to Table 13, a larger GRG value corresponds to better multiple quality characteristics. The importance orders were ranked as welding speed > power > flow rate > pulse shape > laser offset > beam diameter.

**Table 13:** The mean response table of the overall GRG.

Level	Beam diameter	Flow rate	Power	Speed	Laser offset	Pulse shape
1	0.6497	0.6286	**0.7477**	0.5569	**0.6563**	0.6581
2	**0.6540**	**0.6715**	0.5781	0.6668	0.6421	**0.6571**
3			0.6302	**0.7324**	0.6576	0.6409
Delta	0.0042	0.0429	0.1695	0.1755	0.0155	0.0171
Rank	6	3	2	1	5	4

Total mean value of the grey relational grade = 0.6520.

Practically, the parameter A, E, and F showed almost similar values with only a slight difference for the 3rd decimal place. Thus, eights sets of optimal parameter conditions were obtained and are summarized as follows:

**Table:** 

Set 1	A_1_	B_2_	C_1_	D_3_	E_1_	F_1_
Set 2	A_1_	B_2_	C_1_	D_3_	E_1_	F_2_
Set 3	A_1_	B_2_	C_1_	D_3_	E_3_	F_1_
Set 4	A_1_	B_2_	C_1_	D_3_	E_3_	F_2_
Set 5	A_2_	B_2_	C_1_	D_3_	E_1_	F_1_
Set 6	A_2_	B_2_	C_1_	D_3_	E_1_	F_2_
Set 7	A_2_	B_2_	C_1_	D_3_	E_3_	F_1_
Set 8	A_2_	B_2_	C_1_	D_3_	E_3_	F_2_

The ANOVA method was utilized to identify the controllable parameters that significantly affect the quality characteristics. The total variability of the GRGs, which was measured based on the sum of the squared deviations, was separated from the total GRG mean into the contributions of each controllable parameter and the error. Subsequently, the importance of the controllable parameter change on the performance characteristics was evaluated using the percentage contribution of each process parameter in the total sum of the squared deviations. The ANOVA results for the GRG values are tabulated in Table 14.

**Table 14:** ANOVA results of the GRG.

Source	DF	Seq SS	Contribution	Adj SS	Adj MS	F-Value	P-Value
Beam diameter	1	0.000147	0.03%	0.000602	0.000602	0.07	0.792
Flow rate	1	0.015540	2.96%	0.015540	0.015540	1.83	0.189
Power	2	0.165939	31.61%	0.146520	0.073260	8.65	0.002∗
Speed	2	0.151735	28.90%	0.147062	0.073531	8.68	0.002∗
Laser offset	2	0.004485	0.85%	0.004501	0.002250	0.27	0.769
Pulse shape	2	0.000749	0.14%	0.000749	0.000374	0.04	0.957
Error	22	0.186363	35.50%	0.186363	0.008471		
Total	32	0.524958	100.00%				

∗Significant at 5% level.

The ANOVA results in Table 14 indicate that the laser power and welding speed recorded the highest effect on the laser-welded samples with a percentage contribution of 31.61% and 28.90%, respectively. From the P-values at 0.05 significance level, the two parameters have statistically significant effects on the GRG with the laser power demonstrating the most significant parameter on both the quality characteristics. In contrast, the beam diameter, flow rate, laser offset, and pulse shape had a statistically insignificant effect on both the quality characteristics. The findings were similar to that of Aminzadeh *et al.* [38], who also found that the beam power and welding speed were the most influential parameters controlling the weld geometry and mechanical properties of the weld joint.

Furthermore, Bransch *et al.* [27] affirmed that the pulse shaping showed no major influence on the weld dimensions or weld quality of the conduction-mode welds. Although pulse shape I tend to decrease the weld diameter and crater area of the keyhole-mode welds, it showed negligible effect on other measures of size or quality. Conversely, the diameter, penetration, and melt area of the keyhole-mode welds made with pulse shape II were consistently greater than those of pulse shape I. Alternatively, the welds formed in pulse shape II exhibited the greatest crater areas with the least porosity.

Besides, the confirmation experiment was carried out under the optimal welding parameter setting and levels, as previously determined. Table 15 presents the confirmation experiment results using the optimized controllable variables. The A_2_B_2_C_1_D_3_E_3_F_1_ (shown in bold) was identified as the optimal controllable parameters, which corresponds the beam diameter = 0.8 mm (level 2), flow rate = 8 L/min (level 2), power = 0.6 kW (level 1), welding speed = 2.5 mm/s (level 3), laser offset = 0.2 mm (level 3), and pulse shape = type I (level 1). The weld strength was found to increase 38.98% from 236 to 328 MPa, while the weld width was reduced by 7.96% from 1.13 to 1.04 mm.

**Table 15:** Result of the confirmation experiment under optimal settings.

	Level	Weld strength (MPa)	Weld width (mm)	GRG
Initial setting	A_2_B_1_C_1_D_2_E_1_F_3_	236	1.13	0.9168
Prediction	A_2_B_2_C_1_D_3_E_3_F_1_			0.8280
Optimum Setting	A_1_B_2_C_1_D_3_E_1_F_1_	267	1.08	0.8479
	A_1_B_2_C_1_D_3_E_1_F_2_	216	1.11	0.8384
	A_1_B_2_C_1_D_3_E_3_F_1_	320	1.04	0.8539
	A_1_B_2_C_1_D_3_E_3_F_2_	269	1.07	0.8444
	A_2_B_2_C_1_D_3_E_1_F_1_	275	1.08	0.8565
	A_2_B_2_C_1_D_3_E_1_F_2_	224	1.11	0.8470
	**A**_**2**_**B**_**2**_**C**_**1**_**D**_**3**_**E**_**3**_**F**_**1**_	**328**	**1.04**	**0.8625**
	A_2_B_2_C_1_E_3_F_3_F_2_	277	1.07	0.8530

Following the application of the selected optimal controllable variable settings, the predicted optimal GRG showed that D_3_ (welding speed) and followed by C_1_ (laser power) were the most significant parameters. Based on Eq. (16), the calculated predicted GRG was 0.8280, while the confirmation experiments under the optimal setting of A_2_B_2_C_1_D_3_E_3_F_1_ using Eq. (18) was $0.6232\leq {\hat{\gamma }}_{cgg}\leq 1.0328$ at 95% CI. Additionally, the predicted GRG of the confirmation experiment under the optimum condition was 0.8625 at 95% CI, which improved by 4.17% from the predicted mean value. In short, the results showed that the integrated CRITIC and GRA-based Taguchi method demonstrated a significant enhancement of weld quality in Nd:YAG laser welding.

## 5 Conclusion

This paper demonstrated the implementation of integrated multi-criteria function CRITIC and GRA-based Taguchi method to evaluate the optimal combination of the input process parameters comprising the beam diameter, flow rate, laser power, welding speed, laser offset, and pulse shape, for simultaneous low weld width and high weld strength in laser beam welding process. Based on the evaluation and validation of the experimental results via ANOVA, the conclusions of this study were summarized as follows:

(1) The optimal conditions at A_1_B_2_C_1_D_3_E_3_F_1_ comprising the beam diameter = 0.6 mm, flow rate = 8 L/min, power = 0.6 kW, welding speed = 2.5 mm/s, laser offset = 0.2 mm, pulse shape = I achieved the maximum weld strength. Meanwhile, the optimal conditions at A_2_B_1_C_2_D_1_E_3_F_1_ comprising the beam diameter = 0.8 mm, flow rate = 3 L/min, power = 1 kW, welding speed = 1 mm/s, laser offset = 0.2 mm, pulse shape = I recorded the minimum weld width. The measured response values (weld strength and weld width) were based on the confirmatory test results within a 95% CI.
(2) The CRITIC method efficiently estimated the weight fractions for the weld strength and weld width, which were 0.4157 and 0.5843, respectively.
(3) The ANOVA of the GRG results showed that the laser power has the most dominant impact on the total variation, followed by welding speed.
(4) The optimal welding parameter combination of A_2_B_2_C_1_D_3_E_3_F_1_, which corresponds to beam diameter = 0.8 mm, flow rate = 8 L/min, laser power = 0.6 kW, welding speed = 2.5 mm/s, laser offset = 0.2 mm, and pulse shape = I, achieved the simultaneous maximum weld strength and minimum weld width. The GRG value improved by 4.17% from the predicted mean value within a 95% CI of the predicted optimum condition.
(5) The confirmation test verified the effective use of the proposed method to simultaneously improve multiple quality characteristics of the laser beam welding process, in particular the weld strength and weld width. Under optimal conditions, the weld strength and the weld width were enhanced from 236 to 328 MPa and from 1.13 to 1.04 mm (38.98% and 7.96%), respectively.

## Declarations

### Author contribution statement

Teerapun Saeheaw: Conceived and designed the experiments; Performed the experiments; Analyzed and interpreted the data; Contributed reagents, materials, analysis tools or data; Wrote the paper.

### Funding statement

This research was funded by Faculty of Technical Education, 10.13039/501100007345King Mongkut's University of Technology North Bangkok (KMUTNB) Contract FTE-2564-03.

### Data availability statement

Data included in article/supp. material/referenced in article.

### Declaration of interests statement

The authors declare no conflict of interest.

### Additional information

No additional information is available for this paper.
